# Exploring how patient involvement is enacted in the Swedish national system for knowledge-driven management - “work as imagined and work as done”

**DOI:** 10.1186/s12913-026-14266-y

**Published:** 2026-03-06

**Authors:** Christina Petersson, Sylvia Määttä, Boel Andersson Gäre, Göran Henriks, Henrik Ånfors, Ylva Nilsagård

**Affiliations:** 1 Jönköping academy for Improvement of Health and welfare, Jönköping University, Jönköping, Sweden; 2 Qulturum – Center for Learning and Innovation Region Jönköping County, Jönköping, Sweden; 3 Institute of Health and Care Sciences, Sahlgrenska Academy, Gothenburg University, Gothenburg, Sweden; 4 Futurum – the Academy for Research and Education, Region Jönköping County, Göran Henriks, Jönköping, Sweden; 5 Yerevan State University, Yerevan, Armenia; 6 University Health Care Research Centre, Faculty of Medicine and Health, Örebro University, Örebro, Sweden

**Keywords:** Patient participation, Knowledge governance, Evidence-informed practice, Health system governance, National health policy, Knowledge translation, Co-production, Complex systems

## Abstract

**Background:**

Patient involvement occurs at multiple levels of the healthcare system, at micro (patient consultations), meso (team-based collaboration within hospital departments), and macro (shaping policies and governance structures) levels, but research has predominantly focused on the micro level. In Sweden, patient involvement is a core component of the Swedish National System for Knowledge-driven management (NSK), a macro-level initiative. However, little is known about how patient involvement is enacted in practice at this level. This study aimed to explore patient involvement within the Swedish NSK in terms of motives, intentions and suggested progressions. To support the interpretation, we applied a theoretical framework to identify “work” as characterized in four different varieties; work as imagined; work as prescribed; work as disclosed and work as done.

**Methods:**

A qualitative research design was used. Data sources included: (1) steering documents outlining formal policies and strategic goals; (2) interviews with key stakeholders to gather individual experiences and reflections; and (3) non-participant observations from two national working group meetings. Each dataset was first analyzed separately using content analysis and then interpreted through the lens of the Shorrock and Williams framework to enable cross-source synthesis.

**Results:**

Data from documents, interviews, and observations aligned with three of the four varieties of work; Steering documents primarily reflected *Work as Imagined*, describing formal intentions and governance structures for patient involvement. Stakeholder narratives were largely categorized as *Work as Disclosed*, capturing personal interpretations, challenges, and enacted experiences. *Work-as prescribed* was related to the process of collaboration with patient organizations, which also contributed to the recruitment of two patient representatives. Observational data offered limited but insightful examples of *Work as Done*, revealing how patient involvement was performed in practice during meetings.

**Conclusions:**

This study demonstrates the value of combining diverse data sources and applying structured theoretical lens for analyses to better understand how patient involvement is operationalized at the macro level, i.e. how policy intentions are turned into concrete actions, rules and structures. Analyzing observed activities provides essential insight into *Work as Done*, helping to bridge the gap between policy and practice and invites further exploration. Furthermore, the findings contribute to the refinement of the Shorrock and Williams framework by empirically illustrating its applicability in healthcare services research.

**Supplementary Information:**

The online version contains supplementary material available at 10.1186/s12913-026-14266-y.

## Introduction

Internationally, there is a clear trend towards increasing patient involvement in healthcare development and research. This shift aligns with a broader transition from a production-oriented logic to a service-dominant logic, representing a paradigm change from standardized, one-size-fits-all processes to co-created services based on partnerships between patients and healthcare providers [1]. Such partnership requires that patient involvement is grounded in mutual respect and is facilitated through dialogue to make the collaboration meaningful [2]. At the same time, there is a growing demand for healthcare organizations and leaders to support patient and citizen involvement across different levels of the healthcare system [3–6]. Patient involvement manifests across multiple levels of the healthcare system: at the micro level in individual patient consultations, at the meso level through team-based collaboration within healthcare organizations as well as co-design of care processes with patients, and at the macro level in policy formulation and governance structures. Yet research has predominantly focused on initiatives at the micro level. Modigh et al., [7] found that although many studies highlighted the impact of patient involvement at the micro level, few addressed how involvement at the macro level could be realized in practice [7]. Additionally, patient and/or public involvement is a core principle when developing evidence-based guidelines but has not been widely adopted yet [8], which points to the importance of developing structures that include effective and efficient methods of involving patient representatives. A recent study of patient involvement in the Swedish knowledge-driven management system (NSK) demonstrates the importance of patient and next-of-kin involvement in the co-production of national clinical pathways. It also highlights the necessity of appropriate representativeness, supportive working methods, and genuine patient influence on the final outcomes [9]. The Swedish model for NSK is inspired by the Intermountain Healthcare system in U.S, which decades ago created a centralized clinical knowledge-driven management strategy and repository for decision support and care standardization. They used care process models as a core mechanism for codifying evidence-based care pathways and embedded these into practical workflow [10, 11]. At that time, patient involvement was not an explicit part the development but was one of the central principles later when the Swedish NSK was launched. According to Yamazaki et al., [12], there is evidence that clinical care pathways developed within knowledge-driven management frameworks can offer substantial practical benefits, particularly in fostering collaboration and leadership. The clinical pathways promote knowledge exchange among healthcare professionals which is crucial for optimizing patient outcomes and thus support improvements in the quality of care [12]. While knowledge-driven management shows positive effects, by enhancing the ways in which healthcare professionals work, learn and find appropriate knowledge, its potential is still largely untapped. The possibilities for productive use of knowledge-driven management are still largely unused in healthcare settings [13].

Given that patient involvement constitutes a foundational element in the establishment of Sweden’s NSK at the national (macro) level, a deeper exploration of how patient involvement works in practice is warranted. The aim of this study was to examine how patient involvement is conceptualized and enacted within the Swedish NSK, focusing on the motives, intentions, and future progressions of patient involvement at the macro (policy) level. To gain a deeper understanding, the results of the analysis were mapped onto the theoretical framework proposed by Shorrock and Williams [14], which explicitly provides distinct analytical dimensions for capturing a deeper and more systematic understanding of how patient involvement can be enacted in a complex system.

### Theoretical framework

The framework was chosen to gain insight into patient involvement and address gaps between what policy describes and what happens when working groups in the NSK include patient representatives (work in practice, i.e. patient involvement at a national level). Shorrock and Williams’ framework is used not to describe ‘work’ in a narrow task related sense but to conceptualize different representations in practice. In this usage, “work” refers to how patient involvement is imagined in policy, prescribed in steering documents, disclosed in interviews and meetings, and enacted in everyday activities. The value of the framework is that it allows us to systematically distinguish and relate these four forms of ‘work’, rather than merely stating that there is a gap between what is written and what is done. This in turn provides a more fine-grained way of analyzing collaboration around patient involvement, by showing where alignment or misalignment occurs between imagined, prescribed, disclosed and enacted practices.

Other scholars have, for example, used the framework to explore how on-site simulation in maternity care may build organizational resilience and provide high quality of care [15]. The framework has also been used to address system-level factors within healthcare safety investigations concerning blood samples [16].

The concept of *work-as-imagined* and *work-as-done* was first introduced by Hollnagel, [17] to highlight the significant gap between how work is envisioned in policies and procedures and how it is carried out in practice. This emphasized the importance of understanding real-world performance and system resilience in complex socio-technical environments [17]. Shorrock and Williams, [14], further developed and systematized these initial ideas and proposed a framework that conceptualizes human work through adding two varieties: work-as-prescribed and work-as-disclosed. This framework highlights the complexity of human work by moving beyond narrow definitions based solely on formal procedures or direct observations. *Work-as-imagined* refers to how work is conceptualized by different actors ranging from frontline staff to policymakers and within, between, and outside organizations. These mental models often reflect assumptions about how work *should* be performed or how it might be carried out in the future. However, such imaginations are inherently limited and frequently inaccurate, particularly when detached from operational realities. As Hollnagel [18] notes, especially when adverse events occur, the actual course of action often diverges significantly from what was imagined. *Work-as-prescribed* encompasses formalized expectations of how work should be done, typically documented in laws, policies, procedures, checklists, and job descriptions. These prescriptions are usually defined by policymakers or senior organizational members. However, Catchpole and Jeffcott, [19] emphasize that discrepancies between policy and practice are common in healthcare, as it is rarely feasible to prescribe every detail of complex human work. Even in well-understood domains, only the simplest tasks can be fully prescribed. While work-as-prescribed often informs system design and safety assessments, it is frequently supplemented by insights from work-as-imagined or work-as-done [20]. *Work-as-disclosed* relates to what individuals *say* about their work, through interviews, surveys, meetings, or documentation. This version of work may differ from both what is imagined and what is actually done, since what people are willing to disclose is influenced by organizational culture, trust, and perceived fairness. When a culture of psychological safety is present, the alignment between work-as-disclosed and work-as-done is likely to be greater. The last concept is about *work-as-done*, referring to the reality of how tasks are performed in a specific context. It involves dynamic, situated actions characterized by constant adaptation, trade-offs, and decision-making in the face of competing demands, resource constraints, and organizational pressures. This variety of work is often the least visible and most difficult to capture, yet it is the most critical for understanding system functioning and resilience [18]. It is in this domain that practitioners continuously navigate complexity through judgment, improvisation, and local problem-solving. Taking together, these four perspectives provide a nuanced lens for analyzing the gap between formal intentions (policies) and actual practice, particularly relevant when examining how complex concepts such as patient involvement are operationalized in large-scale systems such as the Swedish NSK.

## Methods

The research team, comprising both academically trained researchers, with different professional healthcare backgrounds, and a patient representative, collaboratively established the foundational premises of the study. The inclusion of a patient representative (HÅ) in the research team was important to strengthen the study’s relevance. Drawing on experiential knowledge, the patient representative contributed to refining research questions, interpreting findings from a patient perspective, and ensuring that the results were meaningful and applicable to practice. The dialogue in the research team was initially centered on the rationale for patient involvement, the expectations associated with such engagement, and its potential development over time. Through this process, three core areas of focus were identified to guide the present study: *motives*,* intentions*,* and progression*. To explore these focus areas, a qualitative research design was employed, drawing on methodological principles suitable for capturing complex, context-dependent data [21]. Data collection included a document analysis of relevant steering documents to capture descriptions about patient involvement at macro-level. In addition, individual interviews with key stakeholders and observations of two working group meetings were conducted to provide deeper insight into experiences and interactions at both individual and group levels. Each dataset was analyzed separately and subsequently integrated within the described theoretical framework, allowing for a synthesized interpretation of the findings across all data sources.

### Setting

The present study was conducted within the context of the Swedish NSK for healthcare. Sweden, with a population of approximately 10.4 million, allocates around 11% of its gross domestic product to healthcare. The healthcare system is mainly publicly funded and decentralized, with shared responsibilities between the national government and local authorities. Specialized and approximately two-thirds of the primary healthcare services are managed by 21 regional councils, while 290 municipalities are responsible for social services, including care for the elderly and individuals with disabilities.

In 2017, the regional councils made a national agreement to establish and support a joint structure for the National system for knowledge-driven management (NSK), (in Swedish Nationellt system för kunskapsstyrning). It was guided by a shared vision: *“We count our success in lives and equal health and make each other successful.”* The NSK task is to integrate knowledge support and systematic follow-up with open comparisons of outcomes, data analysis, leadership, and continuous development across organizational borders. The system is supported by the Swedish Association of Local Authorities and Regions (SALAR), which represents and advocates for local and regional governments, to represent them and promote coordination. The ideas of the NSK were inspired by ideas from the Intermountain Healthcare system [22].

The steering group for the NSK serves as a forum for strategic dialogue among key stakeholders, including healthcare directors, SALAR representatives, regional executive officers, and national government representatives. This group is supported by a preparatory body. As of today, the NSK encompasses 26 national program groups addressing major disease areas and several national collaboration groups focusing on thematic areas such as patient safety, quality improvement methods, research and life sciences, and medical technologies. These groups are hosted by the healthcare regions in collaboration with SALAR. Each working group is led by an appointed chairperson with clinical background supported by a designated process leader.

From its inception, the foundational agreement emphasized the importance of collaboration and patient involvement. This principle has since been operationalized at the level of the working groups, where patient and/or next-of-kin representatives collaborate with clinical professionals in the planning and execution of the national healthcare initiatives. Representatives are prepared and supported by the SALAR and by process leaders. The steering group members do not receive hands-on training in collaboration; however, during larger meetings, patient involvement has been the theme of various workshops since the NSK was launched. Patient representatives are well prepared before joining a working group and are informed about the content of relevant documents as well as their expected roles. In the present study, all observed patient representatives had prior experience through their long-term involvement in patient organizations.

### Data collection methods and data analysis

#### Document analysis

A document analysis was conducted in late 2022 to explore how patient involvement is articulated within the national knowledge-driven management system, following the methodology outlined by Bowen [23]. Relevant documents were identified through a systematic search of the publicly accessible website of the NSK. In addition, content from the section on patient involvement on the SALAR website was included. Following email correspondence with the support function at SALAR, one additional document, which was an internal evaluation of the system, was obtained and incorporated into the analysis. Thus, a total of five documents were identified as relevant and included.

Three researchers (CP, SM, and YN) independently reviewed all included documents before engaging in a joint analytical discussion. To structure the analysis, a matrix was developed using Microsoft Excel, with each document represented in a separate row and key analytical components organized into columns. These components included: (1) definition of patient involvement, (2) purpose of the document (e.g., governance, evaluation, recommendation), (3) stated motives for patient involvement, (4) intentions regarding involvement, and (5) descriptions of progression, defined as suggestions for future development. Relevant content corresponding to each analytical component was extracted from the documents and inserted into the matrix. Text excerpts were segmented into smaller analytical units (typically at the sentence level) to facilitate categorization and interpretation. The matrix and resulting interpretations were iteratively discussed among the three researchers until consensus was achieved regarding the classification and meaning of each component, in line with the analytic principles described by Bowen [23].

#### Interviews

**Fig. 1:**
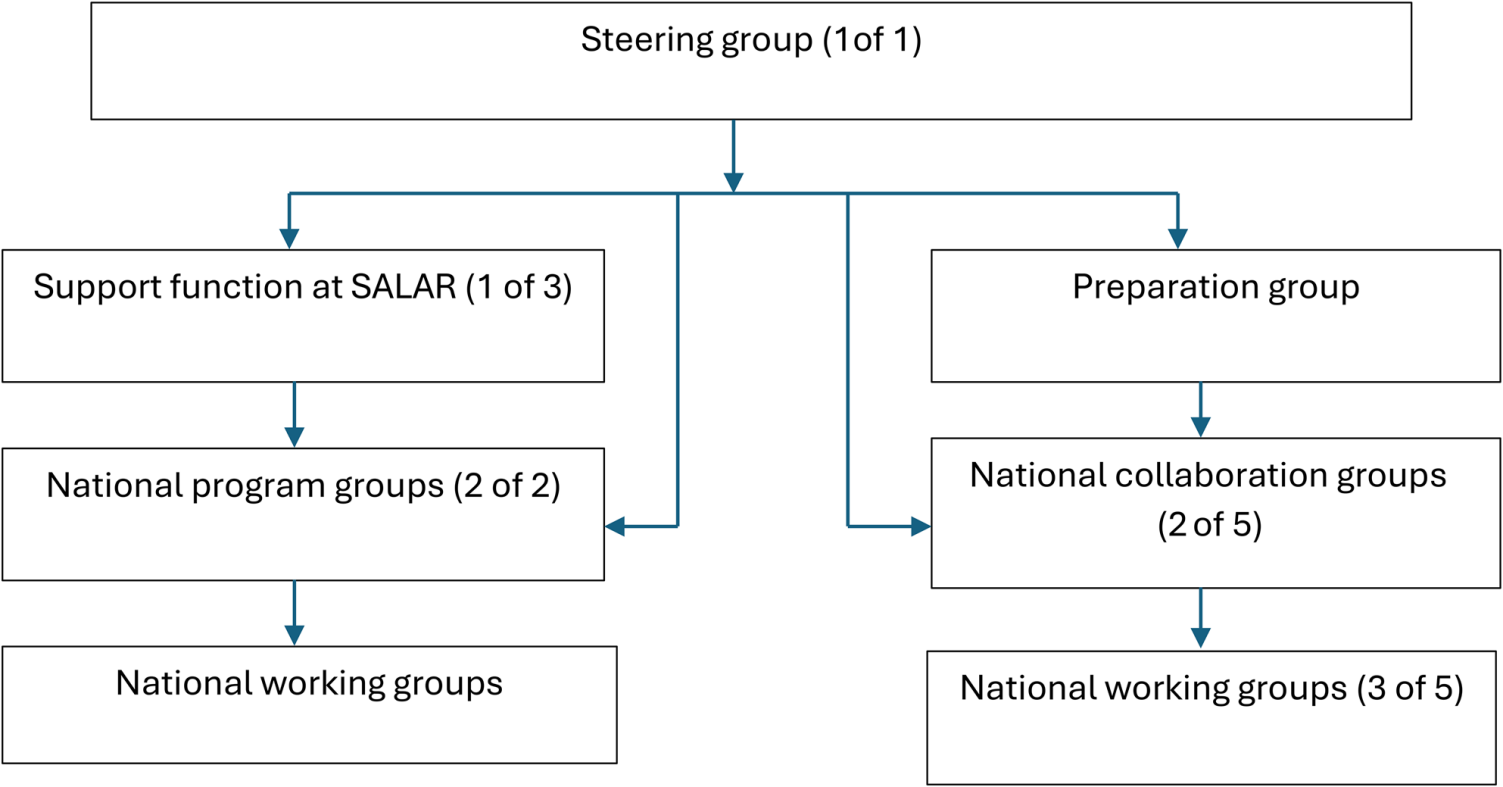
Groups at the national level of the Swedish national system for knowledge-driven management. The numbers in parentheses indicate the number of informants included in the study and the number invited

Individual interviews were conducted with representatives from various stakeholder groups (Fig. 1), selected through strategic sampling to ensure a diversity of perspectives. In total, 16 individuals were invited to participate; nine agreed, three declined, and four did not respond despite two reminders. The final participants consisted of six women and three men, representing a broad range of experience within the NSK context (Fig. 1). Data were collected online via Visiba Care and Zoom between March and June 2023. A semi-structured interview guide, informed by findings from the preceding document analysis, was used to ensure consistency while allowing for flexibility in the exploration of relevant themes. Four main questions guided the interviews: *(1) Patient representatives are currently involved in parts of the system—what are your reflections on this? (2) How would you describe your experience of patient involvement within the NSK? (3) What are your views on patient involvement in the group you represent*,* and why? (4) How do you think the system should evolve to achieve its intended outcomes in the future?* Each main question was supplemented with predefined follow-up prompts, and additional probing questions were used to elicit further depth and nuance. All interviews were audio recorded, lasted approximately 40–45 min, and were transcribed verbatim. The interviews were conducted by SM and YN, both researchers with long experience of qualitative research.

The transcripts were analyzed using deductive content analysis, following the approach described by Elo and Kyngäs [24]. The analysis was guided by the study’s three core areas: motives, intentions, and progression. The process began with repeated readings of two transcripts to achieve familiarity with the data and to generate initial impressions. One researcher (YN) conducted the preliminary coding of these transcripts, identifying and labeling relevant data segments in relation to the three core areas (defined as generic categories). Coding was managed using NVivo software. The initial coding scheme was subsequently reviewed and discussed among three researchers (CP, SM, YN) until consensus was reached. The remaining transcripts were then coded by YN, followed by in-depth discussions among all members of the research team to interpret the content and allocate codes to appropriate generic categories. During this iterative process, some subcategories were refined, renamed, merged, or split based on conceptual clarity and coherence. The final subcategories were derived deductively, based on their alignment with the three generic categories: motives, intentions, and progression. To enhance the trustworthiness of the analysis, the final coding structure and interpretations were discussed and confirmed by all members of the research team.

### Observations

A convenient sampling approach was used by contacting seven national working groups within NSK. Two groups accepted the invitation to participate, while five declined; among the latter, two cited the lack of upcoming meetings, and three did not respond. Prior to data collection, informed consent for observation was obtained from all members of the participating groups. Observations were conducted by two researchers (SM, CP) between October 2022 and January 2023. Each meeting lasted approximately three hours. In the first observation, the meeting included 18 participants, of whom two were patient representatives, and the second observation included 11 participants, of whom one was a patient representative. The researchers were introduced at the start of the meeting by the group chairperson and were seated unobtrusively at the back of the room. In accordance with prior agreement, the observation commenced after the group had conducted one hour of internal work. The meetings were held in person, although one of the groups also included participants who joined remotely. At the time of observation, both groups were in the early stages of developing clinical pathways. According to Bardon et al., [25] observation as a method should be adjusted to the research projects’ special challenges and they recommend an adaptive approach. Therefore, field notes were taken by hand, with a primary focus on verbal interactions, particularly those involving the chairperson and patient representatives. Additional observational elements included room layout, turn-taking, interruptions, body language, and tone of voice. Each researcher transcribed their respective notes independently and subsequently compared them to identify shared patterns and discrepancies. The interpretations were then presented and discussed within the research team. Analytical conclusions were formulated collaboratively and iteratively refined through team discussions until consensus was reached, following the principles of qualitative content analysis [24].

#### Mapping to theoretical framework

Triangulation [26] was employed by integrating the findings generated from the analysis of each dataset and subsequently mapped onto the Shorrock and Williams [14] framework. In this process, a deductive content analysis as described by Elo and Kyngäs [24] was employed. A structured matrix based on the framework was constructed and later used to present the results (see Table 2). During the organizing phase, four researchers (SM, YN, CP, and GH) collaboratively reviewed and mapped the coded data from all sources onto the matrix. This was conducted using a whiteboard to allow dynamic categorization, with iterative adjustments made during discussion until full consensus was achieved. All data segments were successfully categorized within the existing framework, and no modifications to the framework were deemed necessary. Subsequently, the preliminary coding matrix was shared with the remaining members of the research team (HÅ and BAG), who independently reviewed the material. A follow-up discussion with the full research team was held, during which minor clarifications were incorporated to refine the final version of the matrix

### Ethical considerations

The Swedish Ethical Review Authority approved the study (DN 2021-05899-01). All study participants granted consent before the interviews and signed informed consent. We performed the study in accordance with the Declaration of Helsinki.

## Results

The results are divided into four sections, first a description of how motives, intentions and progressions are described in the steering documents. Then, the narratives about these three core areas are outlined. To give insights into how the work is done, the observations are analyzed. The results from the three datasets are then mapped into Shorrock & Willams [14] framework “varieties of human work”.

How motives, intentions and progressions about patient involvement are described in steering documents.

Four of the five analyzed documents articulated explicit motives for patient involvement. These motives were framed within the overarching aim of improving healthcare quality across six established domains: safety, effectiveness, patient-centeredness, timeliness, efficiency, and equity. Achieving this aim was described as a contingent upon the identification of problems and areas for improvement through insights derived from lived experience: knowledge uniquely accessible via patient involvement. The documents emphasized the necessity for reciprocal learning between patients and healthcare professionals, underlining the value of both forms of knowledge as equally important when designing care. Such collaboration was described as requiring fundamentally new ways of working (see Table 1).

All five documents outlined the intention to prioritize patient involvement within the NSK. Patient involvement was described as essential for the sustainable development of future healthcare. The documents called for structured and robust processes that enable patient involvement in central decision-making at the macro level. It was emphasized that knowledge support should be co-produced with input from various actors, including patients, users, next-of-kin, and patient organizations, at the individual, operational, and system levels. Such inclusivity was presented as foundational for high-quality, person-centered services. Concrete recommendations included the presence of at least two patient representatives in each working group and the provision of financial reimbursement for their contributions (see Table 1).

Progressions (future-oriented directions) related to patient involvement were identified in four of the five documents. These included recommendations for continued development of structured dialogue between SALAR’s support functions and patient organizations. An increased focus on healthcare quality and clinical outcomes, particularly as mechanisms to promote equity, was viewed as an area in which patient involvement could be further leveraged. Continued exploration of patient perspectives at the macro level was also advocated, with encouragement to draw on existing collaborative models between patient organizations and regional or municipal healthcare entities (see Table 1).

**Table 1:** Examples of codes from document analysis and illustrating citations

Generic categories	Codes (also mapped to Shorrock & Willams [14] framework in Table 3)	Citations from documents
Motives– why include patient involvement	Identify problems and improvement areas Learn from each other Adjust to quality domains	“When the patients’ experiences, knowledge and perspective are included in the development, it contributes to making healthcare more knowledge-driven, equal and resource-efficient, which in the long run leads to better health for all” (Source: Routine document)
Intentions– how to achieve patient involvement	Develop future healthcare Central decision-making Patient involvement in all levels Two representatives Reimbursed for contribution	“It is important to highlight that the vision “Together” includes both patients, next-of-kin and healthcare staff” (Source: Routine document)
Progressions -what directions lead towards patient involvement	Increase equity Increase quality Strengthen patient involvement at all levels Continuous dialogues	“The patients have influence in all parts of the knowledge-driven management system” (Source: Development report)

### How motives, intentions and progression are described in narratives

#### Motives

Motives were described as “why including patient involvement” (Table 2). *Focusing on patient perspectives for relevance* appeared, which was about understanding more deeply the process for the patient within the process of care. Including patient representatives within the NSK system was seen as supporting the development of the care pathways and could support the possibility to identify gaps in the care provided. The motive for adding patient representatives to the working groups was relying on components that promote co-production, illustrated by this quote:” If *you don’t have patients with you in some way*,* you could forget who we do this for*,* and then I think we are losing it”* (Informant 8).

The second subcategory was described as a manifesto *about patient involvement*. This was exemplified by understanding the purpose and conditions for being involved as a patient representative. The context within the NSK must be considered. The group that is supposed to work together should be clear about the objectives from the start. Then, the anchoring to patient organizations or to patient representatives need some structure. Another aspect was the insight into the complex task that the system put on patient representatives, that the system demands quite advanced knowledge to be able to represent in a working group. It could be exemplified: *“how we anchor our work with patients…that is an opportunity written in [steering documents] … to have patients or patient representatives in the NPOs or NSG or that the work should be reviewed in some regular way”* (Informant 3).

#### Intentions

How to achieve patient involvement was divided into three subcategories (Table 2). First, we found *balancing between individual*,* group and organizational issues*. This was illustrated by the competence to conceptualize, and to problematize concerning different patient perspectives. There was seldom or never a question about the professional representative’s legitimacy, whether they were able to be representatives for their professions or not, therefore there could be a risk of an imbalance between different representatives. Another possible risk that needed to be considered and handled if it occurred was if the patient representatives from a strong patient organization were selected, and these people used the opportunity to push through their organization’s central issues rather than taking general patient perspective. This was illustrated in the quote: “*If you come from a patient organization*,* then you have the strategic agenda of that organization*,* that’s what you have then*,* and it doesn’t cover the entire patient perspective in the disease area”* (Informant 1).

The second subcategory was *finding ways to collaborate*, which was about learning new ways to collaborate with patient representatives. This was exemplified by exploring how to work in the process, define what to be expected and deliver in the “end-product”. The groups all representatives can have the same mandate, if they find an equal relationship between each other, which was seen as a prerequisite in the collaboration process. *“There are a lot of different ways of working and a lot of leading the working group forward in a wise way*,* and there needs to be support so that these perspectives can meet”* (Informant 6).

*Providing support to enhance contributions* was the third subcategory. One example was the education provided to patient representatives; another was supporting them to understand their role within the working group. Building networks among patient representatives and sharing experiences between those working in different groups was seen as important steps on how to strengthen patient involvement. Also, the documents about the NSK were mentioned, where it is outlined how to build support for each representative, illustrated by this citation: “*we could be better at supporting them [patient representatives] in being curious and exploring*,* so that they in turn can carry the information into the contexts they are in”* (Informant 2).

#### Progression

This category describes directions that lead towards patient involvement and were divided into three subcategories describing future steps on how to strengthen patient involvement (Table 2). *Suggesting future possibilities* were exemplified by how to build reference groups or forming workshops to gain a deeper understanding of family perspectives. Patient representatives who were invited to other groups within the NSK suggested that there should be more opportunities to explore patient perspectives in different ways. The aim was not to discuss patient perspectives as a separate topic, but to highlight this in relation to specific issues as they arose. Another example was to have annually structured discussions with patient organizations on a national level as illustrated in this quote: *“I can see the need for representatives in*,* for example*,* NPOs*,* but it depends on which issues are to be handled. It is more important to have the right perspective for each issue than to have a representative who checks it off”* (Informant 4).

Additionally, *challenging current hierarchical structures* was another progression described. To this date, the NSK encompasses a different service logic in comparison to other national initiatives. Bureaucratic and hierarchical structures were mentioned, although these logics are commonly needed in such a big system. To change that kind of dynamic takes time and effort. One way to challenge the structure could be to inverse the relationship regarding the numbers of patient representatives, i.e. that patient representatives would be in the majority and the professional representatives in minority. Illustrated by the suggestion in this quote: *“a group like this that would have a majority of patients then*,* a pretty big step and then patients are also - so it’s hard to be a patient and break this ground*” (Informant 7).

Finally, *increasing transparency* was found to be an important step for future directions. The overall purpose of the NSK was to improve and support the equity of care provided for all citizens in Sweden. To accomplish this, it is important to build trust in the groups where patient representatives are involved. *“Because we are so keen that they feel that we are there for them*,* so we may not always dare to ease the pressure*,* so it can take time to build that security in a group so that you can also say - but what do you think? What do you mean?”* (Informant 6). Another way to reach transparency could be to change representations in the groups after some time, to extend the offer to other representatives to be involved. Also, the healthcare system in Sweden is paid by taxes from citizens, and therefore, is operating on behalf of the population which was a strong argument for being more transparent about the NSK.

**Table 2:** Description of the content of each generic category: motives; intentions: progressions and codes for each subcategory

Generic categories	Subcategories	Codes (also mapped to Shorrock & Willams [14] framework in Table 3)
**Motives** – why including patient involvement	Focusing on patient perspectives for relevance	Understanding more deeply the process; identify gaps in care
	Manifesto about patient involvement	Understanding purpose and conditions; Clear objectives from the start; Anchoring to patient organizations/patient representatives; Insight into the complex task being a patient representative
**Intentions** – how to achieve patient involvement	Balancing between individual, group and organizational issues	Competence to problematize different perspectives; The legitimacy of healthcare professionals’ representativeness seldom disputable compared to patient representativeness
	Finding ways to collaborate	Learning new ways to collaborate; Define what to expect of the end-product; Equal relationship prerequisites for collaboration
	Providing support to enhance contributions	Education to representatives to support the role; Building support to patient representatives
**Progression** – what directions lead towards patient involvement	Suggesting future possibilities	Build reference groups; Annually structured discussions with patient organizations on a national level; Ability to use selected groups and workshops for specific issues
	Challenging current hierarchical structures	Change logics and dynamics; Inversed relationship regarding numbers of patient and professional representatives
	Increasing transparency	To build trust in the groups where patient representatives are involved

### Enacting patient involvement: observations from working groups

To gain a deeper understanding of how patient involvement is enacted in practice, observations were conducted in two separate working groups within the NSK. Three professional members participated via videoconference, while the rest were physically present, seated around a round table. A large screen at the center of the room displayed the remote participants, ensuring visual contact for all attendees. The two patient representatives sat next to each other. The meeting was facilitated by both a chairperson and a process manager. The chairperson guided the meeting in accordance with the agenda, actively engaging participants by soliciting input from those with relevant expertise. Occasionally, the chairperson and process manager conferred briefly on how to steer the discussion forward. Verbal participation varied among group members, and the chairperson strategically directed questions to individuals to elicit contributions. The patient representatives were addressed on multiple occasions and invited to share their perspectives. One of them responded frequently, while the other mainly communicated through non-verbal cues such as nodding and humming, signaling agreement or attentiveness.

In the second observation, the process manager held a preliminary one-on-one meeting with the patient representative in a separate room. They discussed the formulation and distribution of a questionnaire aimed at collecting experiences from patients with the condition in focus. The dialogue was structured around both open and closed questions posed by the process manager to the patient representative. This parallel meeting occurred while the professional representatives engaged in separate discussions. When the full working group convened, all 11 participants were present around an oval conference table. The chairperson led the meeting by inviting members to stand at a podium and present ongoing work. Following each presentation, a few comments were made by other attendees. While the patient representative was present, they were not initially invited to contribute. However, when a general question about patient representation arose, the patient representative chose to speak, articulating their role and perspective.

These observations highlighted several dynamics of patient involvement during meetings. In the first observation, the presence of two patient representatives, one more verbally active—did not seem to cause an imbalance. In the second observation, questions raised by other members about the purpose of involving a patient representative placed the individual in a challenging position. Nonetheless, the patient representative responded by articulating the value of their role, which was reinforced by the process manager, who contextualized the purpose and potential outcomes of co-production.

Notably, the one-on-one meeting between the patient representative and the process manager created a space for more in-depth dialogue, enabling the patient to express views that may not have emerged in the larger group setting. Across both observations, when patient representatives were directly addressed, either by the chairperson or the process manager, they became more actively engaged and contributed with their lived experiences. When provided with space, they also offered feedback on other participants’ contributions. However, their input was not always acknowledged, and in some cases, it was dismissed or not integrated into subsequent discussions. Overall, the active facilitation by the chairperson and process manager was critical for promoting equitable participation and fostering conditions conducive to patient involvement.

### Understanding patient involvement within the NSK according to “varieties of human work”

In this paper, work is defined as prioritizing, producing and launching clinical pathways or other knowledge support documents for healthcare, in collaboration between healthcare professionals and patient representatives.

Codes derived from the document analysis and the narratives were systematically organized and mapped onto the four varieties of human work described in Shorrock’s and Williams (2026) model (Table 3). Codes from the document analysis are marked with a capital “D” in parentheses, while those from the interview narratives are denoted with a capital “I.” The analyzed results from observations are presented in a separate column (Table 3).

To synthesize the results, we found that data from both the documents and the narratives were represented across three of the four varieties of work in the model: Work as Imagined, Work as Prescribed, and Work as Disclosed. As anticipated, most of the document content aligned with Work as Imagined, reflecting formal intentions, strategic ambitions, and governing ideas about patient involvement. In contrast, the narratives predominantly corresponded to Work as Disclosed, capturing individual reflections and practical insights from those engaged in the process. Unsurprisingly, only data from the observations were linked to Work as Done, providing rare but valuable insights into the actual, situated practices within working group meetings. Given that participants in the working groups held varying roles—such as chairpersons, process managers, professional representatives, and patient representatives, the presentation of the observed data is further structured according to these distinct roles, to clarify how responsibilities and actions differed within the practical context of the NSK.

To summarize, **motives** were related to the underlying rationale for patient involvement. In “work as imagined,” motives were described in terms of identifying areas for improvement and fostering mutual learning. In “work as prescribed,” motives were oriented toward aligning with existing quality domains and anchoring participation within organizational structures. “Work as disclosed” revealed insights into the complexity of the patient representative role and the imbalance in perceived legitimacy between professional and patient representatives. Observations (“work as done”) demonstrated that patient representatives actively contributed by sharing lived experiences and articulating the broader relevance of their involvement, while chairpersons and process managers facilitated inclusive dialogue.

**Intentions** referred to the goals and relational strategies underlying collaborative efforts. Documents and interviews emphasized inclusive decision-making and creating equal prerequisites for collaboration. Prescriptions focused on structural enablers such as financial compensation and educational support. Disclosures indicated the emergence of new collaborative practices, while observations confirmed respectful interactional dynamics, such as turn-taking and the directed inclusion of patient voices.

**Progression** captured aspirations for systemic development. Imagined and prescribed dimensions expressed ambitions to increase equity, change power dynamics, and enhance transparency. Disclosed work highlighted ongoing efforts to establish dialogue forums and build trust in representative roles. Observational data supported these trends through the visible actions of stakeholders aiming to facilitate meaningful patient engagement.

**Table 3:** Motives, Intentions and progression mapped into shorrock & williams model varieties of human Work. D=Documents, I=Interviews

	Work as imagined	Work as prescribed	Work as disclosed	Work as done (Observations)
**Motives** (Focusing on patient perspectives for relevance/ Manifesto about patient involvement)	Identify problems and improvement areas (D) Learn from each other (D) Understanding the process deeply (I) Clear objectives from the start (I)	Adjust to quality domains (D) Understanding purpose and conditions (I) Anchoring to patient organizations/patient representatives (I)	Identify gaps in care (I) Insight into the complex task being a patient representative (I)	Patient representatives share lived experiences to highlight consequences for patients as a group. Chairpersons ensured inclusive and structured meetings. They facilitated meaningful involvement of all participants, including patient representatives. The process manager ensured that the value of patient involvement was realized and created conditions for deeper dialogue and understanding.
**Intention** (Balancing between individual, group and organizational issues/ Finding ways to collaborate/ Providing support to enhance contributions)	Develop future healthcare (D) Central decision-making (D) Patient involvement in all levels (D) Competence to problematize different perspectives (I) Define what to expect of the end-product (I) Equal relationship prerequisites for collaboration (I)	Two representatives (D) Reimbursed for contribution (D) Education to representatives to support the role (I) Building support for patient representatives (I)	The legitimacy of healthcare professionals’ representativeness is seldom disputable compared to patient representativeness (I) Learning new ways to collaborate (I)	Patient representatives responded orally to questions and used non-verbal cues (humming and nodding) to signal engagement. Chairpersons led the discussions and allocated speaking turns. The process manager explained and supported the rationale for co-production and clarified expected value and outcomes.
**Progression (**Suggesting future possibilities/ Challenging current hierarchical structures/ Increasing transparency)	Increase equity (D) Increase quality (D) Strengthen patient involvement at all levels (D) Change logics and dynamics (I) Inversed relationship regarding numbers of patient and professional representatives (I)		Continuous dialogues (D) Build reference groups (I) Ability to use selected groups and workshops for specific issues (I) Annually structured discussions with patient organizations on a national level (I) To build trust in the groups where patient representatives are involved in (I)	Patient representatives contributed to shaping the discussion by combining lived experience with more abstract reflections. The chairperson maintained a process that moved toward more balanced involvement. The process manager organized a separate meeting that enabled deeper discussion and richer input from the patient representative

## Discussion

The present study contributes to the knowledge on patient involvement at the national level when groups prioritize, produce and launch clinical pathways, a topic where research is scarce [8]. Three distinct data sources were utilized to examine how the motives and intentions behind patient involvement are enacted in practice, and to explore future recommendations from individuals with experience of working within the NSK. The framework proposed by Shorrock and Williams [14] was employed to illustrate how motives, intentions and progressions could be aligned to the four perspectives on work suggested in the framework i.e. *work-as-imagined*, *work-as-prescribed*, *work-as-disclosed*, and *work-as-done.* There were no observed linear or sequential relationships between the four dimensions when the mapping was made, which is a pattern that aligns with how the framework is described. This non-linearity underscores the complexity of real-world change processes, where shifts in thinking and behavior often occur in parallel, or inconsistently across different organizational levels and roles [27].

The analysis indicated that the steering documents primarily serve to delineate what should be performed and delivered, rather than fostering reflection or promoting an understanding of diverse perspectives related to service provision. Overly detailed steering documents are rarely helpful; the capacity for adaptation and flexibility is essential [28]. The findings also revealed notable gaps between the different perspectives in the framework of varieties of human work. Given that these four conceptualizations of human work are interrelated, we propose that the ability to shift perspectives may support the advancement of patient involvement within the Swedish NSK. One challenge with the steering documents is the risk of them being overly prescriptive or “cookbook-like,” leaving little room for necessary adaptation when translated into practical settings. It is essential that such documents allow for contextual flexibility to support effective interpretation and use in real-world practice. “Work-as-done” often includes activities shaped by situational factors, requiring continuous adaptation, and decisions under competing demands and organizational pressures. Representatives (chairpersons and process leaders) have not been trained specifically in how to collaborate. The documents describe motives and intentions when having patient representatives integrated into the group, but how it is realized is left to be explored by the groups. We emphasize that working methods that actively support the integration of patient perspective, as well as the establishment of a learning-oriented climate are essential components for meaningful patient involvement. Allocating time for such deliberations is likely to enhance the quality of patient involvement and, consequently, maximize its potential impact. It is suggested that the SALAR administration could play a facilitative role in supporting these discussions, particularly in instances where process managers or chairpersons may have limited experience in guiding such reflective practices [9]. The assignments within the NSK are not formulated with the expectation that participants in the different groups will “see and understand” multiple perspectives, but rather that they will “do and deliver.” To support the advancement of patient involvement within the NSK, it is essential that participants can shift between the different perspectives and what lies within work-as-imagined and work-as-done. The *work-as-disclosed* involved insights into the complex role of being a patient representative. While the legitimacy of healthcare professionals’ representation was rarely questioned, patient representativeness required continuous dialogue, and learning new ways to collaborate. There is often an underlying mechanism of hierarchy present in the working groups, and hierarchy has impacts on communication because it can dictate and shape what is acceptable to say and by whom [29].

The observational data clearly demonstrated that both the chairperson and the process manager played pivotal roles in guiding the work within each group which is in line with previous research [9]. The relationship and attitudes in groups are among other factors that have been described as important for successful collaboration [30]. Involving patient representatives in the planning and facilitation of working group meetings may enhance the integration and influence of patient involvement. Previous research has explored methods to support a co-produced approach in healthcare service design. In this context, coproduction represents a shift from traditional top-down models toward collaborative partnerships characterized by shared understanding and joint decision-making [31]. We suggest that patient involvement can be strengthened if all group members are provided with the time and space to critically reflect on their own preconceptions and how they interpret the intended outcomes prior to initiating the work. Such preparatory reflection may serve to bridge the gap between *work-as-imagined* and *work-as-done*.

The *work-as-prescribed* was linked to how quality domains were established and the continuous adaptation to prescribed structures and expectations. A clear understanding of the purpose, underlying conditions, and organizational context shaped how patient involvement was operationalized. As Omaghomi et al., [32] point out, there is a complex interplay between healthcare policy and practice, because policies are meant to influence management strategies and the day-to-day operations in healthcare organizations. This points out the crucial role of aligning practices with evolving policies to ensure that the service is effective and relevant [32].

### Methodological considerations

The use of diverse data sources strengthened the study. This use of triangulation led to a comparison of the results from different perspectives, which gave us possibility to achieve a more comprehensive and multifaceted understanding of the patterns observed [26]. Another strength lies in the broad representation among interview participants. Informants were recruited from all levels and types of roles within the NSK, including chairs and process managers with different professional backgrounds. This diversity allowed for a multifaceted understanding of patient involvement and enabled informants to offer valuable suggestions for future improvement. Of 16 individuals that were asked to participate, only nine accepted. This is a limitation and points to the difficulties in performing studies where the informants have high-level positions. If the data collection during the observations had been audio-taped, we could have analyzed the observations in more depth, while it might have been harder to motivate groups to participate in observation if audio-taping was required.

Throughout the analytical process, the multidisciplinary composition of the research team contributed significantly to the study’s depth and rigor [33]. The team’s varied expertise facilitated critical discussion and reflection, helping to interpret the findings from multiple perspectives, especially to have a patient representative as a part of the research team. While this collaborative process was time-intensive and complex, it also ensured that the analysis was both comprehensive and balanced. We believe this has contributed to the robustness and credibility of the study’s findings.

The study examines the Swedish NSK, which may limit the transferability of the findings to other contexts and may be considered a limitation. Still, the identified design principles and lessons learnt may be used to guide other healthcare systems. The NSK is partly informed by international models, such as Intermountain Healthcare in the United States [22], which may enhance its relevance beyond the Swedish context. The inclusion of observational data was essential for capturing work-as-done, an aspect that is often difficult to access through documents and interviews alone. However, a limitation of the study was the restricted number of observations. Despite initial efforts to include several working groups, access was limited, and only two meetings could be observed.

### Practical implications

One way to enhance patient involvement could be to assign a more active role to patient representatives in collaboration with the process manager when developing meeting agendas, thus promoting more meaningful participation and ensure that patient perspectives are embedded throughout the process rather than added as an afterthought. While this study focused on the national level, it is equally important that the findings related to patient involvement are disseminated across all levels of the healthcare system, particularly at the local level, where clinical pathways are intended to be adopted and put into action.

## Conclusions

While patient involvement is relatively straightforward to articulate and discuss in theory, challenges remain to make the translation into work in practice. This highlights the need for further studies on how preparation of groups early in the process can help close the gaps between stated intentions and actual practices. Moreover, the studied groups were in early stages of their group development process, which probably influences the roles adopted by members and how these roles manifest in practice in the beginning. Nonetheless, it is of value to document and analyze the observed activities, as it contributes to a deeper understanding of work as done, i.e. “what happens” in practice. This study also advanced the knowledge of the applicability of the Shorrock and Williams framework. The analysis of data from diverse data sources into the theoretical framework increased the opportunity to synthesize and understand the interconnectedness of different perspectives of patient involvement on the macro system.

## Supplementary Information

Below is the link to the electronic supplementary material.

Supplementary Material 1

## Data Availability

The data associated with the paper are not publicly available but are available from the corresponding author on reasonable request.
