# Supplementary material for: Exploring how patient involvement is enacted in the Swedish national system for knowledge-driven management - “work as imagined and work as done”
Source: BMC Health Serv Res. 2026 Mar 6;26:483. doi: 10.1186/s12913-026-14266-y (PMC13064297; doi:10.1186/s12913-026-14266-y)
Supplement: Supplementary file 1 — Supplementary Material 1 [file 12913_2026_14266_MOESM1_ESM.pdf]

Supplementary materials

A semi-structured interview guide, informed by findings from the preceding document analysis, was used to ensure consistency while allowing for flexibility in the exploration of relevant themes. Four main questions guided the interviews:

(1) Patient representatives are currently involved in parts of the system—what are your reflections on this?

(2) How would you describe your experience of patient involvement within the KMS?

(3) What are your views on patient involvement in the group you represent, and why?

(4) How do you think the system should evolve to achieve its intended outcomes in the future?

Each main question was supplemented with predefined follow-up prompts, and additional probing questions were used to elicit further depth and nuance.
